# The Use of Neuroimaging for Predicting Sumatriptan Treatment Response in Patients With Migraine

**DOI:** 10.3389/fneur.2022.798695

**Published:** 2022-01-31

**Authors:** Jr-Wei Wu, Pi-Yi Lai, Yung-Lin Chen, Yen-Feng Wang, Jiing-Feng Lirng, Shu-Ting Chen, Kuan-Lin Lai, Wei-Ta Chen, Yu-Te Wu, Shuu-Jiun Wang

**Affiliations:** ^1^Department of Neurology, Neurological Institute, Taipei Veterans General Hospital, Taipei, Taiwan; ^2^College of Medicine, National Yang Ming Chiao Tung University, Taipei, Taiwan; ^3^Institute of Biophotonics, National Yang Ming Chiao Tung University, Taipei, Taiwan; ^4^Department of Radiology, Taipei Veterans General Hospital, Taipei, Taiwan; ^5^Brain Research Center, National Yang Ming Chiao Tung University, Taipei, Taiwan; ^6^Department of Biomedical Imaging and Radiological Sciences, National Yang Ming Chiao Tung University, Taipei, Taiwan

**Keywords:** migraine, sumatriptan, hippocampus, prediction model, brain MRI

## Abstract

**Objectives:**

To identify the neuroimaging predictors for the responsiveness of patients to sumatriptan and use an independent cohort for external validation.

**Methods:**

Structuralized headache questionnaire and 3-Tesla brain magnetic resonance imaging were performed in migraine patients. Regional brain volumes were automatically calculated using FreeSurfer version 6.0, including bilateral amygdala, anterior cingulated cortex, caudate, putamen, precuneus, orbitofrontal cortex, superior frontal gyri, middle frontal gyri, hippocampus, and parahippocampus. A sumatriptan-responder was defined as headache relief within 2 h after the intake of sumatriptan in at least two out of three treated attacks. We constructed a prediction model for sumatriptan response using the regional brain volume and validated it with an independent cohort of migraine patients.

**Results:**

A total of 105 migraine patients were recruited, including 73 sumatriptan responders (69.5%) and 32 (30.5%) non-responders. We divided the migraine patients into derivation (*n* = 73) and validation cohorts (*n* = 32). In the derivation cohort, left hippocampal volume was larger in sumatriptan responders (responders vs. non-responders: 3,929.5 ± 403.1 vs. 3,611.0 ± 389.9 mm^3^, *p* = 0.002), and patients with a larger left hippocampal volume had a higher response rate to sumatriptan (>4,036.2 vs. ≤4,036.2 mm^3^: 92.0 vs. 56.3%, *p* = 0.001). Based on the findings, we constructed a prediction model using the cutoff value of 4,036.2 mm^3^, and we found that patients with a left hippocampal volume >4,032.6 mm^3^ had a higher response rate to sumatriptan than those with a left hippocampal volume ≤4,032.6 mm^3^ (84.6 vs. 42.1%, odds ratio [OR] = 7.6 [95% confidence interval = 1.3–44.0], *p* = 0.013) in the validation cohort.

**Conclusion:**

Our study showed that left hippocampal volume is helpful to identify sumatriptan non-responders. This proof-of-concept study shows that left hippocampal volume could be used to predict the treatment response to sumatriptan in migraine patients.

## Introduction

Migraine is a common and disabling neurological disorder that affects 9–15% of the general population (1–3). Currently, migraine treatment can be classified into acute and preventive therapies, and acute treatments can be categorized as migraine-specific and non-specific (4, 5). Triptans, which are 5-HT_1B/1D_ receptor agonists, are widely used migraine-specific medications to abort acute migraine attacks (6). Even though generic products have emerged, sumatriptan is still the most widely prescribed acute treatment medication for migraine (7, 8). Additionally, clinical trials and post-marketing experience have shown its efficacy and tolerability since the introduction of sumatriptan in the 1990s (9, 10). According to current evidence and real-world experiences, ~30% of migraine patients are non-responders to triptans, and individual responsiveness to triptans is variable (11). To date, the variability in the treatment response is not fully understood (12), and only a few studies have identified the predictors for triptan response in migraine. Current evidence showed that a lower pretreatment pain severity and a higher polygenic risk score were associated with a better response to triptans (7, 8). An early study suggested that triptans' efficacy is less optimal after a patient develops allodynia, but new controlled studies have shown conflicting results (9, 10). Regarding the neuroimaging predictors, no study directly identified structural or functional neuroimaging predictors for sumatriptan response in migraine. On the other hand, the neuroimaging predictor for preventive therapies for migraine has been identified. In chronic migraine, the iron deposition in the periaqueductal gray matter could be used for outcome prediction for onabotulinumtoxinA injection (11). Also, another study found responders to onabotulinumtoxinA injection have cortical thickening in the right primary somatosensory cortex, anterior insula, left superior temporal gyrus, and pars opercularis than non-responders (12). Currently, neuroimaging can be used to differentiate migraine from other primary headache disorders and certain brain regions associated with headache frequency, severity, and long-term outcomes after preventive therapies (13, 14). Hence, this proof-of-concept study hypothesized that neuroimaging could help predict the treatment outcomes of sumatriptan in migraine patients.

## Methods

We retrospectively analyzed the medical records, headache questionnaires, and neuroimaging of patients with migraine who visited the headache clinics of Taipei Veterans General Hospital (TVGH) between January 1, 2015, and December 27, 2017. The included patients should be able to complete the headache questionnaire, and the patient's medical records should be done by board-certificated neurologist specialized in headache medicine.

### Inclusion and Exclusion Criteria of Migraine Patients

The inclusion criteria were as follows: (1). The patient's headache fulfilled the International Classification of Headache Disorders (ICHD-3) criteria for migraine with or without aura, and the headache diagnosis was made by headache specialists; (2). Patients aged between 20 and 49 years; (3). Patients who completed the headache questionnaire; (4). Patients who had used sumatriptan to treat their migraine; (5). Patients who were able to report their treatment response to sumatriptan; and (6). Patients who were able to undergo magnetic resonance imaging (MRI) examinations without contraindications. The exclusion criteria were as follows: (1). Patients with underlying hypertension, diabetes mellitus, cerebrovascular diseases, epilepsy, or other neurodegenerative disorders; (2). Patients who had a history of traumatic brain injury or concussion; and (3). Patients who had been diagnosed with psychiatric disorders were excluded, including major depressive disorders, bipolar disorders, anxiety disorders, or schizophrenia.

### Measures of Sumatriptan Response

All migraine patients participated in a semistructured interview at subsequent visits, which included questions about their response to sumatriptan, the timing of sumatriptan use, and usage of concomitant medications with sumatriptan. A sumatriptan responder was defined as patients with a decrease in headache intensity from moderate or severe to none or mild within 2 h after the intake of sumatriptan, in at least two out of three treated attacks (15–17). Patients with concomitant usage of acute medications other than sumatriptan were excluded from this study to ensure that the treatment responses came purely from sumatriptan.

### Headache Frequency and Severity

In this study, we retrospectively analyzed items of the headache questionnaire, including headache frequency (headache days per month) and the Migraine Disability Assessment Score (MIDAS) questionnaire. The MIDAS questionnaire is widely used in clinical studies and controlled trials in headache medicine for analyzing migraine-related disability in a 3-months period (18, 19). The total score of the MIDAS questionnaire is the sum of five items, including the number of days of missed work/school, reduced productivity at work/school, missed household work, reduced productivity in household work, and missed family and/or social activities.

### Brain Neuroimaging

All participants underwent whole-brain MRI using the same 3.0 T magnetic scanner (Discovery MR750 scanner, GE Healthcare, United States). Acquisition of T1-weighted images was based on 3D-FSPGR and AX-BRAVO sequences with the following parameters: repetition time = 9.384 ms, echo time = 4.036 ms, slice thickness = 1 mm, flip angle = 12°, and matrix size = 256 × 256 × 172 *mm*^2^ using 3D-FSPGR protocol; repetition time = 9.184 ms, echo time = 3.68 ms, slice thickness = 1 mm, flip angle = 12°, and matrix size = 256 × 256 *mm*^2^ using AX-BRAVO protocol. Both 3D-FSPGR and AX-BRAVO were gradient-echo imaging sequences from GE Healthcare suitable for brain volume calculation, and regional brain volumes calculated from automated segmentation of T1-weighted structural images are reliable measures within the same scanner platform, even after upgrades (20).

### Structural Data Processing

After imaging acquisition, preprocessing steps were conducted for better quality and creditability for subsequent analysis to measure the cortical morphological features. The first approach was to correct the head orientation to avoid any motion artifacts by making the AC-PC line congruent with the y-axis by using ART (the acpcdetect program in automatic registration toolbox, https://www.nitrc.org/projects/art). All the images were resized to 1 mm^3^ isotropic voxel with a size of 256 × 256 × 256. Second, bias field correction was performed to remove the inhomogeneity of images by using N4 Bias Field Correction in Advanced Normalization Tools (ANTs). Finally, skull stripping was performed by using HD-BET, which applies artificial neural networks as processing algorithms. Automated brain volume measurements were subsequently conducted using FreeSurfer version 6.0, which is open-source software for processing and analyzing human brain MRI images. The cortical volumes (mm^3^) of the region of interest (ROIs) associated with migraine and analgesic effects were calculated, including the bilateral amygdala, anterior cingulate cortex, caudate, putamen, precuneus, orbitofrontal cortex, superior frontal gyri, middle frontal gyri, hippocampus, and parahippocampus (13, 21, 22).

### Statistics

Comparisons of demographics and clinical profiles between derivation and validation cohort were analyzed by using chi-square or *t*-tests as appropriate. Also, the differences in demographics and clinical profiles in responders and non-responders were analyzed by using chi-square or *t*-tests as appropriate. In the derivation cohort, Bonferroni's correction for multiple comparisons was applied in the comparison of the 20 ROIs between responders and non-responders (corrected for 20 pairwise comparisons: *p* < 0.05/20 = 0.0025). The significant variables were examined by using a classification and regression tree in order to obtain bivariate cutoff values for maximal sensitivity and specificity (23). A chi-square has been applied to compare response rates to sumatriptan between two sides of the cutoff value in the derivation and validation cohorts. The validation of the prediction model in both derivation and validation cohorts was considered exploratory; hence, we used *p* < 0.05 as the significance threshold. All statistical analyses were conducted with IBM SPSS (version 22.0).

### Ethics

The study protocol was approved by the Institutional Review Board of Taipei Veterans General Hospital (2020-03-005AC).

## Results

### Demographics

**Figure 1:**
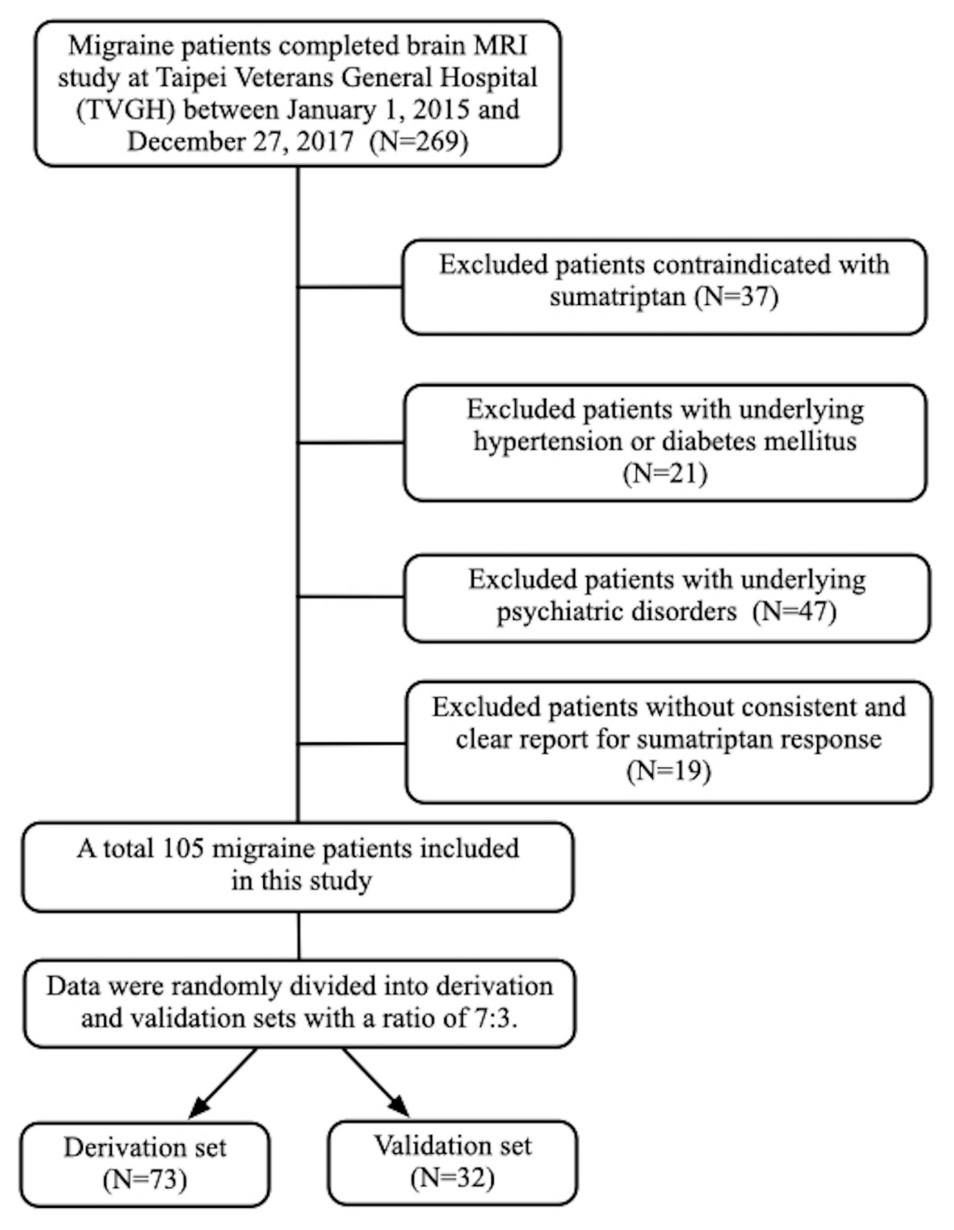
Study schematic flow chart.

A total of 105 individuals with migraine (77 female and 28 male) were included in this study. Among them, 73 were sumatriptan responders (69.5%) and 32 (30.5%) were non-responders (Figure 1). The mean age of the study population was 33.2 (standard deviation [SD] = 8.3]) years. The prevalence of aura was 30.5%, and chronic migraine (CM) accounted for 25.7% of the participants. The derivation and validation sets were randomly divided into at a ratio of 7:3, and there was no difference in demographics between the derivation and validation cohorts, as shown in Table 1.

**Table 1:** Demographics and descriptive statistics of potential confounding factors between the derivation and validation groups.

**Variables**	**Derivation group**	**Validation group**	*p value* ^*^
	**(*N* = 73)**	**(*N* = 32)**	
Age, mean (SD), years	33.4 (9.3)	32.6 (7.8)	0.669
Sex, No. (%)			
Men	16 (21.9%)	12 (37.5%)	0.098
Women	57 (78.1%)	20 (62.5%)	
Prevalence of migraine with aura	23 (31.5%)	9 (28.1%)	0.732
Prevalence of chronic migraine	21 (28.8%)	6 (18.8%)	0.284
Headache frequency	7.9 (7.3)	8.4 (5.3)	0.736
MIDAS	28.9 (30.4)	20.1 (17.0)	0.130
Sumatriptan responder	68.5%	59.4%	0.370
Total intracranial volume	1,537,205.7 (149,894.7)	1,529,646.7 (158,269.3)	0.816
Sequence of brain MRI			
3D-FSPGR	8 (11.0%)	3 (9.4%)	0.806
AX-BRAVO	65 (89.0%)	29 (90.6%)	

* *Results were considered significant by p < 0.05*.

### Potential Confounding Factors of Responders and Non-Responders

Regarding the demographic factors, there are no differences between responders and non-responder in age (mean [SD] years for responders vs. non-responders: 33.4 [9.7] vs. 33.5 [8.7], *p* = 0.960) or sex (responders: 12 males and 38 females; non-responders: 4 males and 19 females, *p* = 0.529). Also, there were no differences in the clinical profiles between responders and non-responders, including prevalence of aura (responders vs. non-responders: 30.4 vs. 32.0%, *p* = 0.894), chronic migraine (CM) (responders vs. non-responders: 26.1 vs. 30.0%, *p* = 0.732), MIDAS (responders vs. non-responders: 29.4 [32.3] vs. 27.8 [26.6], *p* = 0.839), or headache frequencies (mean [SD] headache days per month for responders vs. non-responders: 7.7 [7.1] vs. 8.4 [7.9], *p* = 0.678) (Table 2).

**Table 2:** Demographics and clinical profiles of responders and non-responders in the derivation and validation group.

	**Derivation group (*****N*** **=** **73)**			**Validation group (*****N*** **=** **32)**		
**Variables**	**Responders**	**Non-responders**	*p value* ^*^	**Responders**	**Non-responders**	*p value* ^*^
	**(*N* = 50)**	**(*N* = 23)**		**(*N* = 19)**	**(*N* = 13)**	
Age, mean (SD), years	33.4 (9.7)	33.5 (8.7)	0.960	32.2 (8.4)	33.2 (7.1)	0.719
Sex, No. (%)						
Men	12 (24.0%)	4 (17.4%)	0.529	9 (47.4%)	3 (23.1%)	0.170^**^
Women	38 (76.0%)	19 (82.6%)		10 (52.6%)	10 (76.9%)	
Prevalence of migraine with aura	16 (32.0%)	7 (30.4%)	0.894	6 (31.6%)	3 (23.1%)	0.605^**^
Prevalence of chronic migraine	15 (30.0%)	6 (26.1%)	0.732	4 (21.1%)	3 (23.1%)	0.893^**^
Headache frequency	7.7 (7.1)	8.4 (7.9)	0.678	6.9 (4.0)	9.2 (6.2)	0.166
MIDAS	29.4 (32.3)	27.8 (26.6)	0.839	17.8 (12.2)	23.4 (13.7)	0.23
Total intracranial volume, mm^3^	1,554,551.5 (158,291.6)	1,499,497.4 (124,722.1)	0.115	1,567,413.4 (179,437.5)	1,474,449.3 (104,185.8)	0.104
Sequence of brain MRI						
3D-FSPGR	5 (10.0%)	3 (13.0%)	0.703	2 (10.5%)	1 (7.7%)	0.790
AX-BRAVO	45 (90.0%)	20 (87.0%)		17 (89.5)	12 (92.3%)	

* *Results were considered significant by p < 0.05*.

** *p-value calculated by linear-by-linear association*.

### Regional Brain Volume and Sumatriptan Response (in the Derivation Cohort)

Among the 20 ROIs, the left hippocampal volume was larger in the sumatriptan responders (responders vs. non-responders: 3,929.5 [403.1] vs. 3,611.0 [389.9] mm^3^, *p* = 0.002) (Table 3). Using the classification and regression trees (CRT), we obtained a cutoff value of 4,036.2 mm^3^. By using the chi-square test, we found patients with a larger left hippocampal volume (> 4,036.2 vs. ≤4,036.2 mm^3^) had a higher response rate to sumatriptan (92.0 vs. 56.3%, *p* = 0.001) in the derivation cohort (*n* = 73). We further explored the possible confounding effects on hippocampal values, and we found that hippocampal volume on both sides did not correlate with headache frequency (Left: Pearson's *r* = 0.069, *p* = 0.561; Right: Pearson's *r* = 0.107, *p* = 0.368) or MIDAS (Left: Pearson's *r* = 0.052, *p* = 0.664; Right: Pearson's *r* = 0.189, *p* = 0.110).

**Table 3:** GMV of ROIs (mm^3^) of responders and non-responders in the derivation group.

	**Derivation group (*****N*** **=** **73)**			**Validation group (*****N*** **=** **32)**		
**Variables**	**Responders**	**Non-responders**	*p value* ^*^	**Responders**	**Non-responders**	*p value* ^*^
	**(*N* = 50)**	**(*N* = 23)**		**(*N* = 19)**	**(*N* = 13)**	
**Left side**						
Amygdala	1,512.8 (240.0)	1,470.8 (217.4)	0.477	1,541.7 (204.0)	1,468.5 (177.8)	0.303
Anterior cingulated cortex	4,534.4 (652.5)	4,673.7 (645.3)	0.398	4,627.2 (798.7)	4,536.8 (328.8)	0.703
Caudate	3,373.6 (822.2)	3,164.9 (773.3)	0.308	3,312.0 (964.1)	3,012.9 (508.5)	0.264
Putamen	4,980.6 (675.5)	4,786.3 (446.9)	0.213	5,649.2 (818.3)	3,167.8 (374.4)	0.390
Precuneus	6,158.4 (894.6)	5,906.1 (585.7)	0.221	6,412.9 (1,108.4)	5,174.9 (1,112.0)	0.383
Orbitofrontal cortex	1,114.2 (214.1)	1,038.5 (157.8)	0.124	1,164.1 (221.2)	1,084 (166.9)	0.283
Superior frontal gyri	18,456.4 (2,417.7)	18,337.7 (1,787.0)	0.834	18,835.8 (2,574.1)	18,576 (1,666.4)	0.751
Middle frontal gyri	11,064.5 (1,547.8)	11,056.4 (1,271.4)	0.583	11,270.3 (1,882.9)	16,876.2 (1,749.2)	0.644
Hippocampus	3,929.5 (403.1)	3,611.0 (390.0)	0.002^**^	4,134.4 (401.4)	3,946.3 (370.3)	0.190
Parahippocampus	3,527.4 (555.7)	3,289.1 (473.8)	0.079	3,584.7 (555.8)	3,411.5 (313.9)	0.271
**Right side**						
Amygdala	1,692.9 (264.2)	1,674.0 (243.7)	0.772	1,758.9 (142.5)	1,724.1 (192.6)	0.561
Anterior cingulated cortex	5,842.0 (833.8)	5,826.5 (744.0)	0.959	5,980.2 (761.8)	5,999.5 (725.0)	0.943
Caudate	3,466.4 (762.0)	3,237.2 (737.2)	0.232	3,451.6 (838.0)	3,167.8 (374.4)	0.205
Putamen	5,095.9 (660.1)	4,914.9 (482.7)	0.242	5,360.8 (685.4)	5,174.9 (1,112.0)	0.562
Precuneus	5,722.0 (831.1)	5,462.5 (600.0)	0.184	6,067.2 (966.7)	5,810.8 (885.6)	0.452
Orbitofrontal cortex	976.1 (225.6)	1,030.2 (1,787.0)	0.344	1,055.8 (250.0)	1,056.5 (289.8)	0.994
Superior frontal gyri	16,990.3 (2,243.9)	16,563.1 (1,695.2)	0.426	17,558.6 (2,273.9)	18,576.1 (1,712.5)	0.369
Middle frontal gyri	9,630.2 (1,595.3)	9,483.2 (1,321.4)	0.701	9,663.0 (1,531.8)	9,820.5 (692.3)	0.697
Hippocampus	4,036.1 (434.4)	3,820.2 (430.5)	0.052	4,136.9 (396.7)	4,073.7 (356.1)	0.648
Parahippocampus	3,469.0 (564.2)	3,510.4 (672.9)	0.785	3,298.9 (561.3)	3,375.3 (366.9)	0.670

* *p-value calculated by t-test*.

** *The results were considered significant by p < 0.05*.

### Predicting Sumatriptan Response by Regional Brain Volume

**Figure 2:**
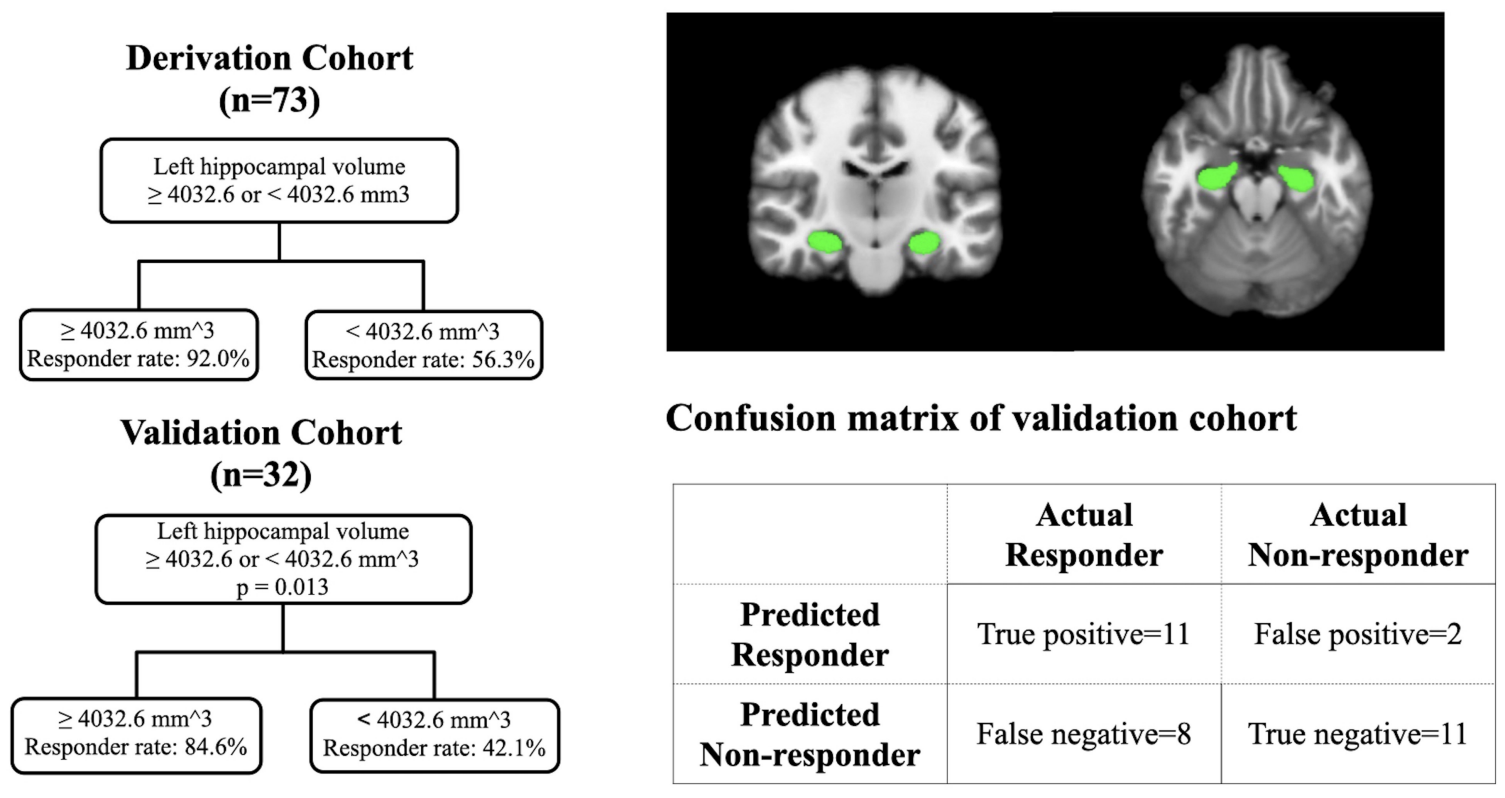
The prediction model in the derivation cohort and validation cohort with the visualization of hippocampus.

Based on the results from the derivation cohort (*n* = 73), we used a cutoff value of 4,032.6 mm^3^ to construct a prediction model by using a classification and regression tree (Figure 2). The validation cohort (*n* = 32), which had no differences in demographics or clinical profiles between the derivation and validation, has been used to examine the prediction model (Table 1). In the validation cohort (Figure 2), patients with a left hippocampal volume >4,032.6 mm^3^ had a higher responder rate than those with a left hippocampal volume ≤ 4,032.6 mm^3^ (>4,032.6 vs. ≤4,032.6 mm^3^: 84.6 vs. 42.1%, odds ratio [OR] = 7.6 [95% confidence interval = 1.3–44.0], *p* = 0.013), with a high specificity and lower optimal sensitivity (specificity = 84.6%, sensitivity = 57.9%, accuracy = 68.8%).

## Discussion

This study found that sumatriptan responders have a larger left hippocampal volume than non-responders. When applying the prediction model to the independent validation cohort, patients with a left hippocampal volume >4,032.6 mm^3^ had a higher responder rate than those with a left hippocampal volume ≤ 4,032.6 mm^3^ (OR = 7.6). The prediction model has a high specificity (84.6%) but a lower optimal sensitivity (57.9%). Instead of identifying good responders, the left hippocampal volume seems to be more suitable for identifying the “poor responders” to sumatriptan.

There are some studies that have aimed to identify predictors for the treatment response to triptans in migraine patients. One early study in 2004 found that pretreatment pain severity is a reliable predictor for the response to sumatriptan (7). Another recent study used genome-wide association studies and found a higher polygenic risk score for migraine associated with the sumatriptan response, which implies that a higher genetic burden of migraine is associated with a better response to migraine-specific treatment (8). To our knowledge, the present study identified left hippocampal volume as a new predictor for the response to triptans in migraine. However, the exact underlying mechanisms are unknown. One possible explanation is the direct effect of sumatriptan on the hippocampus. Although small amounts of triptan may cross the blood–brain barrier (BBB), sumatriptan has lower lipophilicity than other newer triptans (24). The relatively low brain penetration of sumatriptan is less likely to produce direct effects on the hippocampus (25). Additionally, a human postmortem brain study found that the distribution of sumatriptan-binding sites (5HT_1D_ receptor) is higher in the visual cortex, globus pallidus, and frontal cortex than in the hippocampus (26). Therefore, the association between the hippocampus and sumatriptan response seems unlikely to be attributed to the direct effect on the hippocampus.

The second explanation for our study findings is the “maladaptive theory.” This hypothesis is supported by studies that found that patients with smaller hippocampal volumes may be more vulnerable or have maladaptation to stressful events (27). One brain perfusion study found the activation of the amygdala, brainstem, and hippocampus was associated with the analgesic effect of ibuprofen in tooth extraction, and these regions belong to the descending modulatory pathway (22). Another prospective study combined structural and functional MRI to analyze patients with subacute back pain, which found that patients resistant to treatment have smaller amygdala and hippocampal volumes than those responsive to treatment (28). In menstrual pain, one study found that patients with a hippocampal volume associated with BDNF Val66Met polymorphisms and a smaller hippocampal volume had higher severity of menstrual pain (29). Additionally, studies found that patients with chronic pain conditions, (i.e., fibromyalgia, complex regional pain, and chronic low back pain) had smaller hippocampal volumes (30, 31). Regarding migraine, one study from our group found that a smaller hippocampal volume was associated with poor outcomes, indicating that the “maladaptive theory” could be applied to migraine patients (14). These findings suggest that there are reciprocal interactions between the hippocampus and pain; that is, individuals with an underlying smaller hippocampus may be more maladaptive to headaches or other pain conditions, less responsive to analgesics, and more vulnerable to developing chronic pain disorders. In this study, the “non-responders to sumatriptan” might be considered a maladaptive response to pain from a more vulnerable brain (32). The third explanation for the association between sumatriptan response and left hippocampal volume is the pain memory bias. One study could support this explanation, which found exaggerated remembered pain is not uncommon in patients with chronic low back pain. This phenomenon could be attributed to the shape displacement of the left posterior hippocampus (33). However, whether the biased pain memory could be analogized to the memory of analgesic response warrants further research and is beyond the scope of the present study.

The current study has limitations. First, the smaller hippocampal volume may be due to the aging process. Also, our study protocols did not include tests for cognitive function. Hence, the responsiveness to sumatriptan may have memory or recall bias. Nevertheless, the mean age of the present study population was ~30 years, which is an unlikely population to have cognitive deficits. Second, not responsive to one triptan, (i.e., sumatriptan) is not able to predict the response to other triptans (34), and further study is warranted to analyze the neuroimaging predictors of more than one acute medication for migraine. Third, our study excluded patients more than 50-year-old. Hence, our research findings could not represent the pediatric or elder population. The reason for selecting patients between 20 to 49-year-old is to avoid the measurement of brain volume being confounded by the aging process. Also, migraine prevalence peaks from the age 20s to 50s. The prediction model derived from this age range could represent most migraine patients in clinical settings (35). Fourth, our study design did not adjust for confounding factors, such as age, gender, intracranial volume, or ethnicity. The reason for not adjusting these factors is that our proof-of-concept study aimed to construct a prediction model easily applicable to the general population. Also, a recently-published review article addressed that there is no consensus for which and how many covariates should be adjusted for structural imaging studies and stated that “The current results highlight that the use of covariates has statistical and interpretative ramifications (36).” Fifth, the number of responders and non-responders is different in the derivation cohorts, and the imbalanced training dataset may cause overrepresentation of the majority class. On the other hand, our derivation and validation groups were based on data of consecutive patients, and the proportion of sumatriptan responders is usually higher than non-responders in the migraine population. The consecutive patients could prevent the possible confounding effect from the patient selection process.

## Conclusion

This study found left hippocampal volume associated with the response to sumatriptan in migraine patients, and non-responders tend to have smaller left hippocampal volume. According to the prediction model, patients with left hippocampal volume >4,032.6 mm^3^ had a two-fold higher response rate than those ≤4,032.6 mm^3^ in an independent validation cohort.

## Data Availability Statement

The raw data supporting the conclusions of this article will be made available by the authors, without undue reservation.

## Ethics Statement

The studies involving human participants were reviewed and approved by Institutional Review Board of Taipei Veterans General Hospital (2020-03-005AC). Written informed consent for participation was not required for this study in accordance with the national legislation and the institutional requirements.

## Author Contributions

J-WW, Y-TW, and S-JW had full access to all the data in the study and take responsibility for the integrity of the data and the accuracy of the data analysis. J-WW, P-YL, Y-TW, and S-JW: concept and design. J-WW, P-YL, S-TC, Y-LC, Y-TW, and J-FL: interpretation and analysis of neuroimaging. J-WW, Y-FW, K-LL, W-TC, and S-JW: treatment of all patients. J-WW and S-JW: drafting of the manuscript. J-WW: statistical analysis. J-WW, S-TC, Y-TW, and S-JW: obtained funding. Y-TW, J-FL, and S-JW: administrative, technical, or material support. Y-TW and S-JW: supervision. All authors critical revision of the manuscript for important intellectual content and acquisition, analysis, or interpretation of data. All authors contributed to the article and approved the submitted version.

## Funding

This study was supported in part by grants from Taipei Veterans General Hospital [V108C-105 and V107C-135], the Ministry of Science and Technology of Taiwan [MOST110-2314-B-075-081, MOST 110-2314-B-075-035-MY2, MOST 110-2321-B-010-005, MOST109-2314-B-075-002, MOST 108-2321-B-010-014-MY2, MOST 108-2321-B-010-001, and MOST 108-2314-B-010-023-MY3], the Ministry of Health and Welfare, Taiwan [MOHW 107-TDU-B-211-123001 and MOHW 108-TDU-B-211-133001], the Brain Research Center, and National Yang-Ming University from The Featured Areas Research Center Program within the framework of the Higher Education Sprout Project by the Ministry of Education (MOE) in Taiwan.

## Conflict of Interest

The authors declare that the research was conducted in the absence of any commercial or financial relationships that could be construed as a potential conflict of interest.

## Publisher's Note

All claims expressed in this article are solely those of the authors and do not necessarily represent those of their affiliated organizations, or those of the publisher, the editors and the reviewers. Any product that may be evaluated in this article, or claim that may be made by its manufacturer, is not guaranteed or endorsed by the publisher.
